# The integrated care pathway for managing post stroke patients (iCaPPS^©^) in public primary care Healthcentres in Malaysia: impact on quality adjusted life years (QALYs) and cost effectiveness analysis

**DOI:** 10.1186/s12877-020-1453-z

**Published:** 2020-02-18

**Authors:** Aznida Firzah Abdul Aziz, Nor Azlin Mohd Nordin, Amrizal Muhd Nur, Saperi Sulong, Syed Mohamed Aljunid

**Affiliations:** 1 Department of Family Medicine, Faculty of Medicine, Universiti Kebangsaan Malaysia Medical Centre, Level 14, Preclinical Block, Jalan Yaacob Latiff, Bandar Tun Razak, Cheras, 56000 Kuala Lumpur, Malaysia; 2 Center for Rehabilitation and Special Needs, Faculty of Health Sciences, Universiti Kebangsaan Malaysia, Kuala Lumpur, Malaysia; 3 International Centre for Casemix and Clinical Coding, Universiti Kebangsaan Malaysia Medical Centre, Kuala Lumpur, Malaysia; 4 Department of Health Policy & Management, Faculty of Public Health, Kuwait University, Kuwait, Kuwait; 5 Department of Community Health, Faculty of Medicine, Universiti Kebangsaan Malaysia Medical Centre, Kuala Lumpur, Malaysia

**Keywords:** Post stroke, Integrated care pathway, Cost effectiveness, ICER, Quality adjusted life years (QALY)

## Abstract

**Background:**

The delivery of post stroke care is fragmented even in advanced public healthcare systems, globally. Primary care teams are entrusted to provide longer term care for stroke survivors in most developing countries. The integrated Care Pathway for Post Stroke patients (iCaPPS^©^) was designed to guide primary care teams to incorporate further rehabilitation and regular screening for post stroke complications among patients residing at home in communities, using the shared-care approach, especially in areas with limited access to specialist stroke care services. The iCaPPS^©^ addressed coordination of rehabilitation and screening for post stroke complications which were absent in the current conventional care of patients managed at public primary care healthcentres. This study aimed to evaluate the cost effectiveness and impact of iCaPPS^©^ on quality-adjusted- life-years (QALY) compared with current conventional monitoring at public primary care healthcentres.

**Methods:**

A pragmatic healthcentre-based cluster randomised controlled trial-within trial on 151 post stroke patients from 10 public primary care facilities in Peninsular Malaysia was conducted to evaluate QALY of patients managed with iCaPPS^©^ (*n* = 86) vs conventional care (*n* = 65) for 6 months. Costs from societal perspective were calculated, using combination of top down and activity-based costing methods. The 5-level EQ5D (EQ-5D-5 L) was used to calculate health state utility scores. Cost per QALY and incremental cost effectiveness ratio (ICER) were determined. Differences within groups were determined using Mann-Whitney tests.

**Results:**

Total costs for 6 months treatment with iCaPPS^©^ was MYR790.34, while conventional care cost MYR527.22. Median QALY for iCaPPS^©^ was 0.55 (0,1.65) compared to conventional care 0.32 (0, 0.73) (z = − 0.21, *p* = 0.84). Cost per QALY for iCaPPS^©^ was MYR1436.98, conventional care was MYR1647.56. The ICER was MYR1144.00, equivalent to 3.7% of per capita GDP (2012 prices).

**Conclusions:**

Management of post stroke patients in the community using iCaPPS^©^ costs less per QALY compared to current conventional care and is very cost effective.

**Trial registration:**

Trial Registration number ACTRN12616001322426. Registered 21 September 2016. (Retrospectively registered).

## Background

Post stroke care delivery is disorganised and fragmented even in the best of public health systems across the globe [1, 2]. Developing countries face greater challenges in providing optimal post stroke care when resources are prioritised to providing specialist care services, which may not be accessible to the majority. In general, patients receive treatment during acute phase at hospitals, and will be discharged after a 5–7-day stay [3, 4]. Patients and their caregivers will then have to fend for themselves, based on whatever facilities available in the community. Similarly, for those who did not have access to hospitals during the acute stroke period, will eventually seek out treatment from their primary care provider. As stroke care is multidisciplinary per se, organising care across different care environments becomes a challenge, particularly when healthcare resources are finite. To ensure equity in healthcare service provision in developing countries, where most specialist stroke care services are based in urban areas, shared care approaches with the primary healthcare services becomes a necessity.

In-patient rehabilitation facilities for post-stroke patients within the public healthcare system are few and mostly oversubscribed. Assisted living facilities, which are available in developed countries, are mostly non-existent in developing countries. Out-patient rehabilitation services are available in hospitals in urban and some sub-urban areas but often oversubscribed and unable to provide optimum neurorehabilitation services. The patients’ family and the primary care team in their area are the only sources of continuous support for post stroke patients in developing countries. Therefore, pooling resources and expertise to ensure equity in provision of quality post stroke care beyond geographical and health system-related shortcomings, may be the only solution for developing countries.

In Malaysia, the primary care teams, led by trained Family Medicine Specialists (FMS), have been instrumental in providing post stroke care at public primary care healthcentres in the last two decades. However, the FMS’ faced challenges in coordinating the rehabilitation aspect for stroke survivors at the healthcentres, for those initiated by the tertiary care team or for stroke survivors who present late to the healthcentres [5]. The addition of rehabilitation services at public primary care healthcentres in the last 5 years (i.e. from 2011 to 2012 onwards) has reduced access-related difficulties to some extent. Public primary care healthcentres are now equipped with either Physiotherapy and/or Occupational Therapy services to provide general rehabilitation service for the community. The local clinical practice guidelines for management of stroke [6, 7] did not address the role of the primary care team in provision of longer-term stroke care (i.e. medical and rehabilitation aspects) for the majority of patients who were discharged home and residing in communities which lack access to Specialist Stroke care services.

Hence, realising this need, the integrated Care Pathway for Post Stroke (iCaPPS^©^) protocol was designed to overcome the gap in post stroke care delivery and promote shared care initiatives within the local public healthcare system currently in place. The iCaPPS^©^ protocol was designed by a local panel of specialist stroke care providers and FMS’ from both the Ministry of Health as well as academics cum clinicians based at tertiary university hospitals in the country. The expert panel used current non standardised protocols at their place of practice, evidence-based knowledge as well as recommendations for improvements by the panel within existing public healthcare services. The methodology for iCaPPS^©^ is described in another publication [8]. Rehabilitation intervention and regular screening for post stroke complications were additional features of iCaPPS^©^ compared with conventional care practices at public primary care facilities (Table 1). Utilising current infrastructure and work force within the healthcare system to revamp the current system will require tangible evidence to stakeholders and policy makers to make the much-needed changes.

**Table 1:** Comparison between iCaPPS^©^ and Conventional care management of post stroke patients at public primary care health centres

Assessment / Treatment	Monitoring / screening procedure	Conventional Care	iCaPPS^©^
Stroke Risk factor(s) monitoring or NCD monitoring	Vital signs (blood pressure, pulse rate)	+	+
	Fasting blood sugar	+	+
	HbA_1c_	+	+
	Fasting blood lipids	+	+
	Renal function / eGFR	±	+
Screening for stroke-related complications	Functional status	–	+
	IADL	–	+
	Depression	–	+
	Cognitive assessment	–	+
Rehabilitation intervention / assessment of progress	Physiotherapy	±	+
	Occupational therapy	±	+
	Speech/language therapy	–	+

*eGFR* estimated (calculated) Glomerular Filtration Rate, *IADL* Instrumental Activities of Daily Living

+done on a regular basis, as per schedule, − not done regularly, ± occasionally done

### Aims / hypothesis

The aim of this study was to undertake a cost effectiveness analysis of the implementation of iCaPPS^©^compared to conventional post stroke care from societal perspective, using the Euroqol EQ-5D-5 L. Our hypothesis being that iCaPPS^©^ would not be more expensive than conventional care.

## Methods

The design of the trial was a pragmatic cluster randomised controlled trial-within trial at public primary care healthcentres. The trial was conducted between 1st July 2012 till 31st July 2013.

### Healthcentre selection

Multi-staged sampling was done to select 10 public primary care healthcentres across Peninsular Malaysia which provided post stroke care for patients residing at home in the community. Public primary care healthcentres in Malaysia are inhomogeneous in terms of service provision. Hence, the cluster of healthcentres included were shortlisted based on three main criteria i.e.: (i) the availability of on-site support services at the healthcentre i.e. physiotherapy and/or occupational therapy services (ii) location of the healthcentre which represented selected zones i.e. northern, central, southern and eastern Peninsular Malaysia and (iii) willingness of the FMS and support staff at the healthcentres to participate in the trial. FMS’ were approached using snowballing technique, 3 months before the trial commenced to identify stroke patients among the patients who attended the Non-Communicable Disease (NCD) and general primary care clinics. This was necessary as there was no information available on the incidence or prevalence of stroke patients, either at national or public primary care healthcentre level. Information on longer term stroke patients receiving treatment at each healthcentre had to be manually searched. The healthcentres recruited as study sites were then randomized using coin toss method, assigned to either conventional care or iCaPPS^©^ protocol for post stroke monitoring (5 healthcentres each). No standardized protocol for stroke care existed therefore the different clinics were allotted one of two types of protocols.

### Patient recruitment

**Fig. 1:**
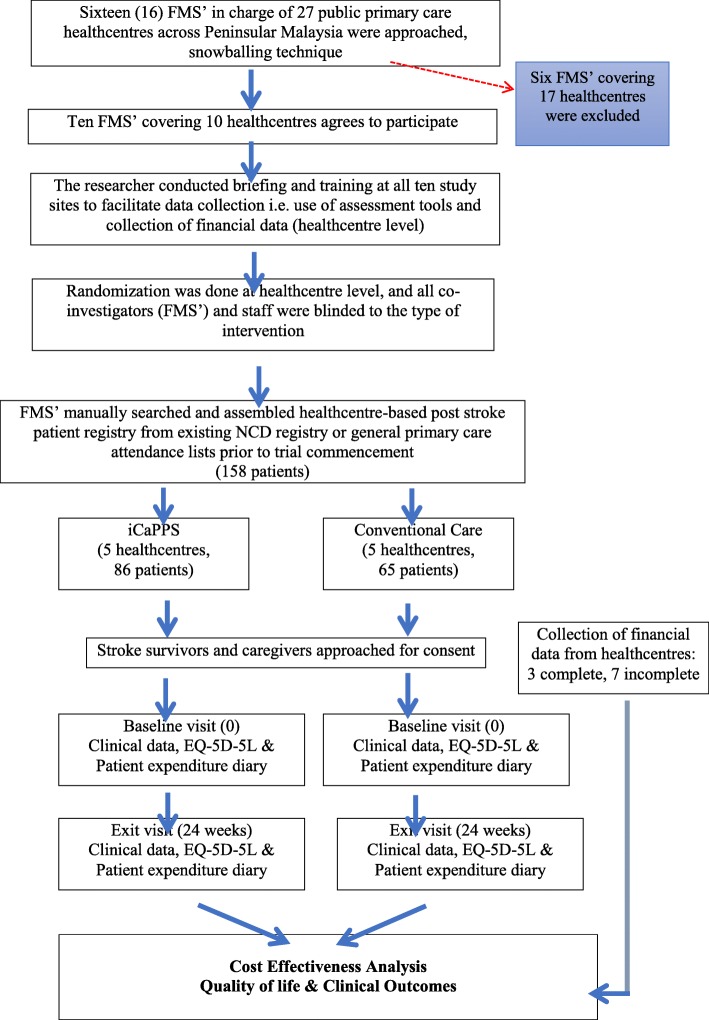
Study Flow Chart

Using the healthcentre-based stroke registry prepared for this study, purposive sampling of patients’ medical records was screened to assess eligibility. Post-stroke patients, either discharged from hospital and / or undergoing treatment at public healthcentres, aged 18 years and above, any type of stroke diagnosed clinically by Neurologist / Physician and / or confirmed radiologically, at least 6 months or more after first or recurrent stroke episode, completed acute stroke treatment and discharged from hospital, completed acute stroke treatment and referred for longer term stroke care at community healthcentres were recruited. Patients who have other neurological diagnosis such as transient ischemic attack, traumatic brain injury, isolated nerve palsies were excluded. Patients and/ or their caregivers were contacted and invited to participate in this study. For aphasic patients, the accompanying caregivers were approached for consent. Written informed consent was also obtained. Patients were given follow up appointments under the FMS (i.e. site investigator) once they consented to participate. Protocol for follow up was conducted as schedule as per conventional care (CC) or iCaPPS^©^. Baseline and exit visit at 6 months were all conducted by the FMS at each healthcentre. For those on iCaPPS^©^ arm, visits were scheduled as per protocol. The researcher visited all study sites to monitor and verify information collected during the trial. Refer Fig. 1 for details.

### Sample size calculation

Considering the constraints in resources for this study, power of study was set at 80%, with the desired group difference in mean EQ-5D-5 L index scores estimated at a rate of 15%. A change in behavioral indicators in the order of 10–15% is recommended as the minimum for target group survey efforts, as attempts to measure changes of smaller magnitudes with adequate precision would exceed available resources [9]. Following this assumption, 65 patients were required on each arm. Due to the lack of studies on long-term outcomes of stroke patients residing at home in community, it was decided that studies which assessed quality of life on patients residing in the community at least 6 months post stroke, and utilised any outpatient facilities was used as guide to calculate sample size of subjects [10–12].

A total of 151 patients were recruited during the trial period (July 2012–July 2013), where patients were monitored for duration of 6 months according to the assigned protocols. The primary outcome of the trial was quality of life of post stroke patients undergoing both programs, using the EuroQoL EQ-5D-5 L questionnaire.

### Economic evaluation

The cost analysis was conducted from a societal perspective, i.e. where all possible costs and benefits to all sectors of society were estimated. In this study, this comprises the healthcare providers, stroke patients and caregivers.

### Cost analysis

This was done following principals determined by Drummond et al. [13]. Provider costs were calculated by estimating capital costs (i.e. building, clinic equipment costing ≥RM500) and recurrent costs (i.e. administration, maintenance, utilities, Staff emolument and benefits, consumables and drugs) incurred to operate the healthcentre. Sources of data came from annual returns, administrative and financial records for year 2012. In all healthcentres, charges for the patients were waived if patients were Malaysians who were retired civil servants, from elderly age group (i.e. at or more than 60 years old) and if patients were registered with the Social Welfare Department as ‘Orang Kurang Upaya’ (OKU) or physically challenged persons.

Patient diaries provided primary data for patient out-of-pocket costs. This included money spent on transportation costs to and from the healthcentre, meals taken during trips, service or clinic registration charges other than at public healthcare facilities, miscellaneous expenditures incurred resulting from stroke complications (e.g. alternative and complementary treatments). Loss of productivity for patient and/ or their caregivers was included. Patients were asked to fill out the diaries during the clinic visits, and the average values were used.

Productivity loss was calculated based on human capital approach- valued according to patients’ and/or caregivers’ reported income per hour. In cases where patients or caregivers refused to disclose their annual gross income for personal reasons, the gross domestic per capita income for 2012 (RM30,956) was used to estimate productivity loss and cost of informal care delivered by housewives or unemployed family members [14, 15]. For stroke survivors who were retirees or pensioners, the reported total annual pension received or the gross domestic per capita income, whichever available, was used to calculate productivity loss. This is based on literature from economic studies have shown that pensions raise productivity [16] and this study was an attempt to estimate monetary value to productivity loss to the community as a result of the stroke. A combination of step-down and activity-based-costing (ABC) methods was employed. For the ABC method, the iCaPPS^©^ protocol was used to guide calculation of cost components. All were added up to provide the total cost. In the ABC method, since the data was not normally distributed, the median (IQR) was used for calculation of costs.

The cost effectiveness analysis in this study utilised the data of treatment outcomes in the form of patients’ reported quality of life scores and health state utilities weights. For healthcentres assigned to iCaPPS^©^ and caregivers who were able to complete the EuroQoL EQ-5D-5 L questionnaires, changes in the utility index values after treatment was directly used as utility scores. For the healthcentres managed under conventional care and for patients with had missing EQ-5D-5 L values, the patients’ functional status i.e. Barthel Index scores was scored by the researcher during monitoring visit, and mapped with EQ-ED utility index values, following the methodology described by van Exel et al. [17]. For other variables, intention to treat principles were applied.

For this study, the QALYs were calculated at baseline (recruitment) and exit visit at week 24. The differences in treatment outcomes (i.e. QALY) between the two programmes were calculated. This was done by multiplying the utility weights with the expected number of remaining life-years following the acute stroke period. The expected number of remaining life-years was obtained from life expectancy tables for stroke survivors generated by Hannerz and colleagues [18] as the morbidity and mortality of stroke survivors were not comparable to that in a normal population.

The incremental cost effectiveness ratio (ICER) was imputed by dividing the differences in cost of the alternative programmes with the differences between outcomes. Finally, a sensitivity analysis was performed to test if any changes in the parameters would change the ICER values and influence conclusions made from the CEA. The trial protocol is summarised in Fig. 1.

Approval to conduct the study was obtained from the Ethics Committee, Faculty of Medicine, Universiti Kebangsaan Malaysia (Research ID GUP-UKM-2011-321) as well as Ministry of Health, Malaysia (Research ID: NMRR-11-1074-10,358).

## Results

### Sociodemographic and clinical characteristics of the post stroke patients

The overall mean age at stroke was 55.8(SD9.8) years. The sociodemographic characteristics of patients in both arms were not statistically different (please refer Table 2). Clinical changes of the patients after 24 weeks are summarized in Table 3.

**Table 2:** Socio-demographic characteristics of patients enrolled according to intervention groups (*N* = 151)

Variables	Subgroups	N (%) or Mean ± SD or Median (IQR)		Test	*p* value
		Conventional Care (*n* = 65)	iCaPPS^©^ (*n* = 86)		
Age at stroke onset, years	≤ 50 years	19 (29.2)	25 (29.1)	Χ^2^ = 2.223,df = 3	0.527
	51–60 years	22 (33.8)	35 (40.7)		
	61–70 years	11 (17.0))	16 (18.6)		
	≥71 years	13 (20.0)	10 (11.6)		
Duration post stroke, years		4.0 (6.0)	1.29 (3.0)	z = − 2.354	0.190
Gender	Male	31 (47.7)	50 (58.1)	Χ^2^ = 1.625, df = 1	0.202
	Female	34 (52.3)	36 (41.9)		
Ethnicity	Malay	39 (60.0)	41 (47.7)	Χ^2^ = 2.258, df = 1	0.133
	Non-Malay	26 (40.0)	45 (52.3)		
Smoker status	Non-smoker	56	78	Fisher’s exact	0.441
	Current smoker	9	8		
Annual income (MYR)		8640 (22572)	15,300 (21000)	Z = -1.904	0.057

**Table 3:** Changes in clinical characteristics of patients enrolled according to intervention (N = 151) after 24 weeks

Variables	Subgroups		N(%) or Mean (SD) or Median (IQR)		Test	*p* value
			Conventional Care	iCaPPS^©^		
Systolic BP (mmHg)	Baseline		130.0 (120.0,143.5)	130.0 (120.0,140,0)	z = −0.172	0.864
	Exit		131.0 (120.0,150.0)	130.0 (120.0,140.0)	z = −0.853	0.394
Diastolic BP (mmHg)	Baseline		76.0 (70.0,82.0)	80.0 (70.0,85.0)	z = −0.929	0.353
	Exit		80.0 (70.0,84.0)	80.0 (70.0,84.0)	z = −0.280	0.780
Total cholesterol (mmol/L)	Baseline		4.98 (4.13,5.73)	4.60 (4.01,5,44)	z = −1.138	0.255
	Exit		4.90 (4.19.5.86)	4.45 (3.90,5.20)	z = −2.067	0.039
LDL-cholesterol (mmol/L)	Baseline		3.2 ± 1.0	2.9 ± 0.9	t = 1.886, df = 137	0.061
	Exit		2.9 (2.2,3.9)	2.4 (2.0,3.2)	z = −2.557	0.010
Triglyceride (mmol/L)	Baseline		1.4 (1.1,2.0)	1.3 (1.0,2.0)	z = −1.683	0.092
	Exit		1.5 (1.1,1.9)	1.4 (1.1,1.7)	z = −1.037	0.300
HDL-cholesterol (mmol/L)	Baseline		1.3 (1.0,4.7)	1.2 (1.0,1.4)	z = −2.038	0.021
	Exit		1.1 (1.0,1.4)	1.1 (1.0,1.4)	z = 0.779	0.436
HbA_1c_ (%)	Baseline		7.2 (6.4,11.2)	6.7 (6.0,7.9)	z = −2.445	0.014
	Exit		7.1 (6.2,10.4)	6.7 (6.0,7.8)	z = −1.927	0.054
Functional status, modified Barthel Index (mBI) scores	Baseline		90 (75,100)	95 (75,100)	z = −0.430	0.667
	Exit		82.1 (75,100)	95 (80,100)	z = −0.400	0.689
Depression screen, PHQ9 scores	Baseline	≥10	6 (9.2)	6 (7.0)	Fisher’s exact	0.793
		< 10	59 (90.8)	80 (93.0)		
	Exit	≥10	1 (1.2)	6 (7.0)	Fisher’s exact	0.240
		< 10	85 (98.8)	80 (93.0)		

### Quality of life, EQ-5D-5L scores

The overall median EQ-5D-5L utility score at baseline is 0.53 (0.40,0.73). Analysis between both groups using Mann Whitney tests showed that there were no significant differences in both EQ-5D-5L scores at baseline and at 6 months (*p* > 0.05). See Table 4.

**Table 4:** Changes in EQ-5D scores among post stroke patients in 24 weeks (N = 151)

Status of EQ5D utilities scores	N(%)	
	Conventional Care	iCaPPS^©^
Improvement	15 (23.1)	31 (36.1)
No change	45 (69.2)	34 (39.5)
Deterioration	5 (7.7)	21 (24.4)

#### Cost analysis

From the total of 10 healthcentres recruited, only three provided complete financial records. Assumptions could not be made to replace the incomplete financial data from other healthcentres as these centres were inhomogeneous in the profile of the staff, location (i.e. urban, suburban or rural), size of healthcentre complex and services available. Hence, patient diaries from patients attending the three healthcentres (iCaPPS^© ^= 51, CC = 24) provided primary data for patient-out-of-pocket expenditures and time taken off work, whenever applicable, for the 24-week duration of the trial. A complete case analysis was conducted for all patients using healthcentre specific financial data at each study site.

#### Total cost of post stroke monitoring programs

The total cost for 6-month post stroke treatment at primary care public healthcentres according to iCaPPS© protocol was MYR790.34 (1172.67) while conventional care was MYR527.22 (370.68) (z = − 3.252, *p* = 0.001). Refer Table 5 for details.

**Table 5:** Cost of post stroke monitoring programmes for 24 weeks’ duration from societal perspective

Variables	Median (IQR)		Mann Whitney test, z	p
	iCaPPS^©^	Conventional care		
Number of clinic visits	6.0 (4.0,7.25)	6.0 (5.0,8.0)	−1.82	0.069
Providers’ cost (MYR)	546.40 (555.69)	329.26 (220.93)	−2.645	**0.008**
Patients’ cost by components (MYR)				
Patients’ loss of income / productivity	18.94 (69.14)	28.64 (77.57)	−1.159	0.247
Caregivers’ loss of income / productivity	29.31 (70.57)	82.39 (70.44)	−2.486	**0.013**
Other Direct costs^a^	120.00 (278.70)	34.00 (104.00)	−3.681	**0.000**
Total patient cost	199.43 (407.87)	123.41 (151.48)	−2.831	**0.005**
Total cost for (MYR)	790.34 (1172.67)	527.22 (370.68)	−3.252	**0.001**

^a^Private clinics and other charges related to treatment for stroke, also includes transport and food and beverage expenses during visit

Figures in bold are statistically signifcant

#### Cost components or cost drivers

In terms of distribution of providers’ costs by components, staff emolument (58.2%) was the highest, followed by drug costs (27.7%), maintenance (4.7%), consumables (3.7%), administration (3.3%), utilities (1.9%) and equipment (0.5%). For patient costs, other direct costs (e.g. costs for treatment from private clinics, charges related to treatment for stroke plus transportation costs, food and beverage expenses during the visits) were the major component (71.3%), followed by caregivers/ productivity loss (16.5%) and patients’ productivity loss (12.2%).

#### Cost per quality adjusted life-years (QALY) gained

The costs incurred in order to gain one QALY via iCaPPS^©^ and conventional care is displayed in Table 6.

**Table 6:** Cost per QALY for post stroke care at community level

Treatment outcomes	QALY Median (IQR)		Outcome gain Median (IQR)	Therapy Cost (MYR)Median (IQR)	Cost per QALY (MYR) (2012 price)
	Pre-treatment	Post-treatment			
iCaPPS^©^	8.55 (6.4)	9.10 (5.9)	0.55 (1.65)	790.34 (1172.67)	1436.98 (USD469.60)
Conventional Care	7.01 (6.1)	7.50 (7.10)	0.32 (0.73)	527.22 (370.61)	1647.56 (USD538.41)

#### Incremental cost-effectiveness ratio (ICER)

This study demonstrated that post stroke care using the iCaPPS© resulted in RM1144 per QALY gained compared to conventional care. This value is equivalent to 3.7% of the average Gross Domestic Product (GDP) per capita for year 2012 (RM30956). ICER values for clinical parameters are also displayed in Table 7.

**Table 7:** Incremental Cost Effectiveness Ration for Clinical Outcomes

Variables	Changes after 24-weeks		Incremental Cost (MYR) (Cost _A_-Cost _B_)	Incremental effectiveness	ICER (MYR per unit reduction or gain)	% GDP
	iCaPPS^©^	Conventional Care				
Systolic blood pressure (mmHg)	0.61	−2.23		2.84	92.65	0.3
Diastolic blood pressure (mmHg)	0.16	−1.12		1.28	205.56	0.7
Total cholesterol (mmol/L)	−0.24	−0.05	263.12	0.19	1384.04	4.5
LDL-cholesterol (mmol/L)	−0.27	− 0.06		0.21	1252.95	4.0
Functional status (BI scores)	1.77	0.94		0.83	317.01	1.0
Quality Adjusted Life Years (QALY)	0.55	0.32		0.23	1144.00	3.7

*Cost*_*A*_ *= MYR790.34, Cost*_*B*_ *= MYR527.22*

*BI* Barthel Index, *GDP* Gross Domestic Per capita income (for 2012: MYR 30956.00), *iCaPPS©* Integrated Care for Post Stroke, *QALY* Quality Adjusted Life Years

#### Sensitivity analysis

In this study, the scenarios, were made on the assumption that the providers’ cost may be altered in situations such as the total number of visits (in 6 months) made to the healthcentres for post stroke care (i.e. for either consultation, laboratory investigations or for prescription refill or combination of reasons), lower ranking personnel were involved in the care provision or the healthcentres did not have facilities for rehabilitation.

The best-case scenario was assumed when post stroke management had resulted in the highest improvement in health status, as per achieved in QALY and clinical outcomes-especially functional status. Hence, the patient and/or the caregiver no longer feels that further rehabilitation (i.e. therapist-led sessions held at healthcentres) would be beneficial, or patients had attained the highest functional ability based on their stroke type and continued to exercise while in their own homes or both parties lack awareness on its benefits i.e. did not pursue rehabilitation therapy. However, these patients and/or their caregivers will continue to seek treatment for the stroke risk factors or co-morbid conditions from the healthcentres.

For the worst-case scenario, the assumption was made on the basis that post stroke management for those who showed potential to benefit from rehabilitation, which had lapsed or had not been initiated after discharge from tertiary care. Hence, patients seen at the healthcentres received full spectrum of care as outlined by iCaPPS^©^ programme, which comprises therapist-led rehabilitation sessions as well as post stroke care monitoring by FMS. Refer Table 8.

**Table 8:** Incremental Cost Effectiveness Ratio (ICER) in scenario-based sensitivity analysis

Variables	iCaPPS^©^			Conventional Care		
	Best case	Base case	Worst case	Best case	Base case	Worst case
Therapy cost (MYR)	757.86	790.34	1308.03	440.48	527.22	546.55
QALY gained	0.55	0.55	0.55	0.32	0.32	0.32
Cost per QALY gained	1377.92	1436.98	2378.24	1376.50	1024.96	1707.97
Incremental Cost (MYR)	317.38	263.12	761.48	–	–	–
Incremental effectiveness	0.23	0.23	0.23	–	–	–
ICER (RM per QALY) (2012 prices)	1379.91	1144.00	3310.78	–	–	–

Currency exchange rate for 2012 is MYR3.01=USD1.00

## Discussion

Evaluation of cost effectiveness between healthcare programs, which deliver post stroke care to patients in the community, is important in order to ascertain if any impact is made on the quality of life of these patients. Clinical outcomes are generally aimed at reaching treatment targets for secondary prevention, and by and large, are similar even in different care environments [19]. In reality, the clinical outcomes do not necessarily translate to improved quality of life although survival is relatively improved with reduction in stroke recurrence. Assessment of quality of life is difficult, as it is highly subjective to the individual. One of the methods which can be employed to measure the health outcome of a patient is to attempt to equate it in monetary terms, i.e. how much money is required to improve the quality of life which is now altered due to illness and disability. The tools used to measure QoL are numerous, and for this study, the Euroqol EQ-5D-5 L was chosen as it facilitated estimations to be made to the patients’ functional status (i.e. Barthel Index scores in situations where these readings were not routinely done for patients at most public primary care healthcentres)^16^. Hence, there is a necessity for a CEA to justify the need for change in any healthcare service delivery. A pragmatic trial-within trial was chosen to evaluate the effectiveness as well as cost-effectiveness of an intervention (i.e. iCaPPS^©^) under real-time conditions, which could be implemented once the program is accepted for monitoring long-term stroke patients in the community [20, 21].

Firstly, our study is the pioneer to determine cost effectiveness of a post stroke service from a societal perspective, and secondly the evaluation of service provision from public primary care facilities. Analysis of costs and outcomes revealed that the cost for post stroke monitoring with iCaPPS^©^ was almost 50% higher than the cost of conventional care. The difference is due to the increased provider’s cost, which was mainly driven by the staff salaries and drugs. However, the outcome of iCaPPS^©^ in terms of cost per QALYs is 12.7% lower than conventional care.

The iCaPPS© programme had increased involvement of the staff at the healthcentre, engaged in doing assessments and screening for stroke-related complications, which are generally overlooked in conventional care. Comparison with other literature is difficult as most CEA for stroke include costs covering healthcare utilisation across different care environments, which are not similar to our study settings. In most literatures, the costs of stroke include acute care in tertiary, hospitalisation costs for inpatient rehabilitation at community rehabilitation hospitals and domiciliary home services. The main cost drivers in these studies were for hospitalisation and nursing home costs [22, 23].

In our study, the patients mainly resided at home after discharge, bypassing long-term admission (institutionalisation) in community rehabilitation facilities or state-funded nursing homes usually described in literatures from developed countries. From the patients’ perspective, our study documented that the loss of productivity of caregivers’ was slightly more than the patients’ themselves (16.5% vs 12.2%). This finding provides objective evidence of economic consequences for the caregiver of stroke patients who reside at home in the community.

An earlier study conducted by our group evaluating the cost for post stroke monitoring at a Specialist outpatient clinic in a teaching hospital [24] the providers’ cost was almost double the costs incurred using iCaPPS^©^ at healthcentres, for same duration of 6 months. (RM990.20 vs RM527.22) (2012 prices). We postulate that this may be due to the type of patients seen at the Specialist outpatient clinic who will comprise of patients who are in earlier phase of stroke recovery rather than those in chronic or long-term stroke and likely to have more complications compared to those seen at primary care facilities. The patients in the early phase of stroke recovery would incur higher costs of drug care and also due to complex care regimes in the initial stages to stabilize. Compared to primary care services, where the majority of patients would be in the long-term or chronic phase, and managed using resources available at primary care healthcentres.

In determining the cost effectiveness of a health intervention, the WHO Commissions on Macroeconomics and Health [25] defined interventions that have a cost effectiveness ratio of less than three times of gross domestic product (GDP) per head as cost effective (i.e. Threshold approach). Our study showed that in terms of cost per QALY gained, after 6 months, the iCaPPS^©^ had lower cost per QALYs than Conventional care (RM1436.98 vs RM 1647.56) and this is further strengthened by incremental cost effectiveness ratio (ICER) for iCaPPS^©^ (RM1,144 per QALY). This is classified as highly cost effective due to it being lower than one gross domestic per capita income for our country (GDP 2012 = MYR30956.00) (Acharya 2003). Hence, our study has demonstrated that using the iCaPPS^©^ protocol at public healthcentres, a cost-effective option is available to deliver quality post stroke care in underserved areas,

The sensitivity analysis conducted for this study was based on current public health care service provision in this country and catered towards the various types of primary care visits for long-terms care of stroke patients who are synonymous with patients requiring chronic care or treatment for non-communicable diseases.

## Limitations

This trial only included public primary care healthcentres, which were mainly located in Peninsular Malaysia, and targeted only patients and caregivers who could access the healthcentres. Cost analysis was limited to only 6 months due to time and funding constraints, although the iCaPPS^©^ was designed to cater for longer periods where stroke survivors continue to visit the public primary care healthcentres for as long as they live. The iCaPPS^©^ screening components cater for longer-term complications which are also associated with ageing (e.g. swallowing, cognitive decline).

Financial data was only available from three out of the 10 primary care public healthcentres. Some of the financial records of the healthcentres were not available on site, and were kept in the District Health Office in charge of several healthcentres within the region. Filing of financial records for each district was inhomogeneous and this made tracing the records difficult. The period taken to trace the financial records alone took 12 months in total, and caused unavoidable delays during the trial. Future studies should address estimation of national unit costs for care at public primary healthcare services according to various healthcentre subtypes to facilitate future economic evaluations.

## Conclusion

Based on the CEA analysis, the iCaPPS^©^ is a very cost-effective method for monitoring post stroke patients who are residing at home, particularly for those accessing longer term care at public primary care healthcentres and warrants consideration for nationwide implementation in public primary care healthcentres.

The evaluation of domiciliary post stroke care services using the iCaPPS^©^ programme is an area which should be explored, to cater for stroke survivors who are bed –ridden or who are unable to access the public healthcentres. Costing for similar services provided by private primary care facilities should also be explored to provide appropriate disbursement rates once the national health-financing scheme comes to fruition.

## Data Availability

The datasets used and/or analysed during the current study are available from the corresponding author on reasonable request.

## Abbreviations

CEA: Cost Effectiveness Analysis
EQ-5D-5 L: EuroQoL EQ-5D-5 L
iCaPPS^©^: Integrated Care Pathway for Post Stroke patients
NCD: Non-Communicable Disease

## References

[1] op Reimer WJM S, DWJ D, Franke CL, van Oostenbrugge RJ, de Jong G, Hoeks S (2006). Quality of hospital and outpatient care after stroke or transient ischemic attack: insights from a stroke survey in the Netherlands. Stroke..

[2] Cameron JI, Tsoi C, Marsella A (2008). Optimizing stroke systems of care by enhancing transitions across care environments. Stroke..

[3] Hamidon BB, Raymond AA (2003). Risk factors and complications of acute ischaemic stroke at hospital Universiti Kebangsaan Malaysia (HUKM). Med J Malaysia.

[4] Nazifah SN, Azmi IK, Hamidon BB, Looi I, Zariah AA, Hanip MR (2012). National Stroke Registry ( NSR ): Terengganu and Seberang Jaya Experience. Med J Malaysia.

[5] Abdul Aziz AF, Mohd Nordin NA, Abd Aziz N, Abdullah S, Sulong S, Aljunid SM (2014). Care for post-stroke patients at Malaysian public health centres: self-reported practices of family medicine specialists. BMC Fam Pract.

[6] Malaysian Society of Neurosciences & Academy of Medicine Malaysia & Ministry of Health Malaysia (2006). Clinical Practice Guidelines. Management of Stroke.

[7] Malaysian Society of Neurosciences Academy of Medicine Malaysia Ministry of Health Malaysia (2012). Clinical Practice Guidelines. Management of Ischaemic Stroke.

[8] Abdul Aziz AF, Mohd Nordin NA, Ali MF, Abd Aziz NA, Sulong S, Aljunid SM (2017). The integrated care pathway for post stroke patients (iCaPPS©): a shared care approach between stakeholders in areas with limited access to specialist stroke care services. BMC Health Serv Res.

[9] Adamchak S (2000). A Guide to Monitoring and Evaluating Adolescent Reproductive Health Programs: FOCUS on Young Adults.

[10] Pickard AS, Johnson JA, Feeny DH, Shuaib A, Carriere KC, Nasser AM (2004). Agreement between patient and proxy assessments of health-related quality of life after stroke using the EQ-5D and health utilities index. Stroke..

[11] Darlington A-SE, Dippel DWJ, Ribbers GM, van Balen R, Passchier J, Busschbach JJV (2007). Coping strategies as determinants of quality of life in stroke patients: a longitudinal study. Cerebrovasc Dis.

[12] Lopez-Bastida J, Oliva Moreno J, Worbes Cerezo M, Perestelo Perez L, Serrano-Aguilar P, Montón-Álvarez F (2012). Social and economic costs and health-related quality of life in stroke survivors in the Canary Islands, Spain. BMC Health Serv Res.

[13] Drummond M, Sculpher M, Torrance G, O’Brien B, Stoddart G (2005). Methods for the Economic Evaluation of Health Care Programmes.

[14] Liljas B (1998). How to calculate indirect costs in economic evaluations. Pharmacoeconomics.

[15] Drummond M (2001). Introducing economics and quality of life measurements into clinical studies. Ann Med.

[16] Dorsey S, Cornwell C, Macpherson D (1998). Pensions and productivity. Pensions and productivity.

[17] van Exel NJA (2004). Scholte op Reimer WJM, Koopmanschap MA. Assessment of post-stroke quality of life in cost-effectiveness studies: the usefulness of the Barthel index and the EuroQoL-5D. Qual Life Res.

[18] Hannerz H, Nielsen ML (2001). Life expectancies among survivors of acute cerebrovascular disease. Stroke..

[19] Ovbiagele B, Drogan O, Koroshetz W (2008). Outpatient practice patterns after stroke hospitalization among neurologists. Stroke..

[20] Schwartz D, Lellouch J (1967). Explanatory and pragmatic attitudes in therapeutical trials. J Chronic Dis.

[21] Buxton MJ, Drummond MF, Van Hout BA, Prince RL, Sheldon TA, Szucs T (1997). Modelling in economic evaluation: an unavoidable fact of life. Health Econ.

[22] Caro JJ, Huybrechts KF, Duchesne I (2000). Management Patterns and Costs of Acute Ischemic Stroke : An International Study. Stroke.

[23] Dewey HM, Thrift AG, Mihalopoulos C, Carter R, Macdonell RA, McNeil JJ (2001). Cost of stroke in Australia from a societal perspective: results from the north East Melbourne stroke incidence study (NEMESIS). Stroke..

[24] Aznida F, Azlin NM, Amrizal M, Saperi S, Aljunid S (2012). The cost of treating an acute ischaemic stroke event and follow-up at a teaching hospital in Malaysia: a Casemix costing analysis. BMC Health Serv Res.

[25] Acharya A, Barendregt J, Birch S, Brock D, Brouwer W, Carande-Kulis V, Edejer T, Baltussen R, Adam T, Hutubessy R, Acharya A, Evans D (2003). Making choices in health: WHO guide to cost-effectiveness analysis.

